# Distinct B subunits of PP2A regulate the NF‐κB signalling pathway through dephosphorylation of IKKβ, IκBα and RelA

**DOI:** 10.1002/1873-3468.12912

**Published:** 2017-12-03

**Authors:** Yoshihiro Tsuchiya, Keiko Osaki, Mayu Kanamoto, Yuki Nakao, Ena Takahashi, Toru Higuchi, Hideaki Kamata

**Affiliations:** ^1^ Laboratory of Biomedical Chemistry Department of Molecular Medical Science Graduate School of Biomedical Science Hiroshima University Japan

**Keywords:** NF‐kappa B, protein phosphatase, protein phosphatase 2 A (PP2A)

## Abstract

PP2A is composed of a scaffolding subunit (A), a catalytic subunit (C) and a regulatory subunit (B) that is classified into four families including B, B′, B′′ and B′′′/striatin. Here, we found that a distinct PP2A complex regulates NF‐κB signalling by dephosphorylation of IKKβ, IκBα and RelA/p65. The PP2A core enzyme AC dimer and the holoenzyme AB′′′C trimer dephosphorylate IKKβ, IκBα and RelA, whereas the ABC trimer dephosphorylates IκBα but not IKKβ and RelA in cells. In contrast, AB′C and AB′′C trimers have little effect on dephosphorylation of these signalling proteins. These results suggest that different forms of PP2A regulate NF‐κB pathway signalling through multiple steps each in a different manner, thereby finely tuning NF‐κB‐ and IKKβ‐mediated cellular responses.

## Abbreviations

**IκB**, inhibitor of kappa B

**IKKβ**, inhibitor of kappa B kinase β

**IKKβKN**, kinase‐negative IKKβ mutant

**NF‐κB**, nuclear factor kappa B

**PPPs**, protein phosphatases

Nuclear factor kappa B (NF‐κB) is a critical transcription factor that regulates many cellular and organismal processes including immune and inflammatory responses, cellular growth and cell survival. The NF‐κB signalling pathway is regulated by the phosphorylation of several proteins including inhibitor of kappa B (IκB) kinase β (IKKβ), an inhibitory protein IκBα and an essential subunit of NF‐κB RelA/p65 1, 2, 3. Binding of cytokines such as TNF‐α to respective receptors leads to the conjugation of ubiquitin to several signalling components and recruits adaptor proteins and kinases 3, 4, 5, 6. These chains of reaction induce IKKβ activation through phosphorylation of two critical serine residues, Ser177 and Ser181, at its activation loop 7, 8. IKKβ in turn phosphorylates IκBα at the N‐terminal serines, Ser32 and Ser36, which leads to ubiquitination at lysine residues Lys21 and Lys22 and IκBα degradation by the ubiquitin‐proteasome system. These reactions result in the nuclear translocation of NF‐κB and binding to its cognate κB sites in the promoters/enhancers of its target genes. IKKβ also phosphorylates serine residues, Ser468 and Ser536, in the C‐terminal transactivation domain of RelA and promotes gene expression through the association of RelA with a transcriptional coactivator, CREB‐binding protein 9, 10, 11.

Aberrant activation of NF‐κB is linked to various diseases such as inflammatory disorders and cancer. Thus, there are numerous regulatory mechanisms at multiple levels to ensure the tight control of NF‐κB activity 1, 2, 3, 4, 5, 6. In particular, dephosphorylation of its protein represents the most important mechanism to downregulate NF‐κB activity. There are three families of protein serine/threonine phosphatases: protein phosphatases (PPPs), metal‐dependent protein phosphatases (PPMs) and DxDxT phosphatases 12, 13. PPP constitutes the largest family, containing several members including protein phosphatase 1 (PP1), PP2A, PP2B and PP5, whereas the PPM family includes PP2C. Several of these, such as PP1 14, PP2A 7, 15, 16, PP2C 17 and PP5 18, are required for dephosphorylation of the activation loop serines in IKKβ, which is a critical step to downregulate NF‐κB. In addition, protein phosphatases dephosphorylate downstream signalling proteins as well. Specifically, it has been reported that PP2B dephosphorylates IκBα 19 and PP2A 20 and PP2C 21 dephosphorylate RelA.

Among these phosphatases, PP2A is the most abundant, constituting approximately 1% of total cellular proteins 22, 23, 24. PP2A is a heterotrimeric complex consisting of a scaffolding (A), regulatory (B) and catalytic (C) subunit. The A and C subunits each are comprised of two possible variants, α and β. The monomeric C subunit is unstable and requires binding to the A subunit to exist as a stable core enzyme AC dimer. The B subunit family consists of four classes including B (B55/PR55), B′ (B56/PR61), B′′ (PR48/PR72/PR130) and B′′′(PR93/PR110)/striatin (Strn). In turn, there are four isoforms of the B class including α, β, γ and δ, five of the B′ class including α, β, γ, δ and ε, three of the B′′ class including α, β and γ, and three isoforms of the B′′′/Strn class including Strn, Strn3 and Strn4. These B subunits associate with the AC dimer to form the holoenzyme trimer. Crystal structure analysis revealed that the B subunits bind proximal to the C subunit active site in the holoenzyme trimer and determine the specificity for substrate proteins 25, 26.

A previous RNAi screen revealed that PP2A plays an important role in the regulation of NF‐κB signalling and identified the core enzyme subunits, Aα, Aβ, Cα and Cβ, as negative regulators of the NF‐κB signalling pathway in mouse astrocytes 16. However, this screen did not clarify whether the B subunit is involved in regulation of NF‐κB activity, nor identify the specific B subunits regulating each NF‐κB signalling step. It is plausible that, as cells usually express multiple isoforms of each B family protein, this failure was a result of complementation with other isoforms of the same family following the simple RNAi screen. In this study, we expressed B subunits together with A and C subunits in cells and investigated the role of these subunits on TNF‐α‐, IKKβ‐ and RelA‐induced NF‐κB activation. This assay revealed that distinct B subunits regulate specific steps of NF‐κB signalling. Dephosphorylation of IKKβ and RelA is mediated by the AC dimer and AB′′′C trimer, whereas dephosphorylation of IκBα is mediated by the AC dimer, ABC trimer and AB′′′C trimer.

## Materials and methods

### Cell cultures, plasmids and transfection

cDNA‐encoding Aα, Aβ, Cα, Cβ, Bα, Bβ, Bγ, Bδ, B′α, B′β, B′γ, B′δ, B′ε, B′′β, Strn and Strn3 subunits of PP2A were amplified from a human cDNA library by PCR. The cDNAs were inserted into pRK‐HA and pRK‐Flag expression vectors. Expression plasmids encoding IKKβ, kinase‐negative IKKβ mutant (IKKβKN) and IκBα have been reported previously 27. Constitutive active IKKβEE mutant of which phosphorylation sites Ser177/181 in the activation loop were exchanged to Glu, the ubiquitination‐resistant IκBαRR mutant of which ubiquitination sites lysine residues Lys21/22 were exchanged to Arg, and the phosphatase‐inactive CαD85N mutant of which Asp 85 was exchanged to Gln were generated using the KOD‐Plus Mutagenesis kit (TOYOBO). Plasmids were transfected into GP2‐293 cells in Opti‐MEM (Invitrogen) using Lipofectamine Plus (Invitrogen) following the manufacturer's instructions. GP2‐293 cells were obtained from Clontech. Cells were cultured in DMEM supplemented with 10% FBS containing 2 mm L‐glutamine at 37 °C.

### Luciferase assay

Nuclear factor kappa B activity was estimated by Dual‐Luciferase Reporter Assay System (Promega) using pNF‐κB and pRL‐TK Luciferase reporter plasmids. After transfection of 0.01 μg NF‐κB reporter plasmids with or without 0.01 μg expression plasmids of various PP2A subunits, IKKβ, IκBα and/or RelA into GP2‐293 cells in collagen‐coated 96‐well dishes, cells were incubated in the presence or absence of 50 ng·mL^−1^ TNF‐α for 18 h. After lysing cells with buffer from the assay system, luciferase activity was analysed following the manufacturer's instructions.

### Immunoblotting

GP2‐293 cells in collagen‐coated 12‐well dishes were transfected with 0.2 μg expression plasmids of various PP2A subunits, IKKβ, IκBα and/or RelA. After 24‐h transfection, cells were washed with PBS and solubilized with buffer A consisting of 20 mm Tris/Cl, pH 7.5, 150 mm NaCl, 1 mm EGTA, 10 mm MgCl_2_, 60 mm β‐glycerophosphate, 1 mm Na_3_VO_4_, 1 mm 4‐amidino phenyl methyl sulfonyl fluoride, 50 KIU·mL^−1^ aprotinin, 20 μg·mL^−1^ pepstatin, 20 μg·mL^−1^ leupeptin, 2 mm DTT and 1% Triton X‐100. After centrifugation at 16 000 × ***g*** for 20 min at 4 °C, the supernatants were used as cell lysates. The cell lysates were subjected to SDS/PAGE and then gel‐separated proteins were transferred to PVDF membranes (Millipore) and subjected to immunoblotting using the SuperSignal West Pico Chemiluminescence System (Pierce). Antibodies used were as follows: anti‐Flag (M2) (Sigma, F‐3165), anti‐HA (Roche, 04‐902), anti‐IκBα (Santa Cruz Biotechnology, SC‐371), anti‐RelA/p65 (Santa Cruz Biotechnology, SC‐372), anti‐phospho‐IκBα (Cell Signaling Technology, 2859), anti‐phospho‐RelA/p65(S468) (Cell Signaling Technology, 3039), anti‐phospho‐RelA/p65(S536) (Cell Signaling Technology, 3033), anti‐PP2A (Cell Signaling Technology, 2039), anti‐PP2A (Epitomics, 1512‐1), anti‐IKKβ (Cell Signaling Technology, 2684) and anti‐phospho‐IKKα/β (Cell Signaling Technology, 2697).

### *In vitro* dephosphorylation assay by PP2A

For the dephosphorylation assay of IKKβ, GP2‐293 cells in 10‐cm dishes were transfected with expression plasmids encoding Flag‐IKKβ, and then phosphorylated IKKβ was recovered from cell lysates *via* anti‐Flag (M2) agarose beads (Sigma). IKKβ proteins were eluted in a buffer containing 20 mm Tris/Cl, pH 7.5, 150 mm NaCl, 2 mm DTT, 1% Triton X‐100 and 3 × Flag peptides (Sigma) from anti‐Flag‐agarose beads with ultra‐free Centrifugal Filter Units (Millipore). For the dephosphorylation assay of IκBα and RelA, cells were transfected with plasmids encoding Flag‐RelA, HA‐IκBαRR and IKKβEE, and then phosphorylated IκBαRR and RelA protein complexes were purified from cell lysates *via* anti‐Flag (M2) agarose beads. PP2A proteins were purified from GP2‐293 cells following transfection with plasmids encoding the Flag‐tagged Aα subunit and HA‐tagged Cα subunit, together with or without HA‐tagged B subunits including Bα, Bβ, Bγ, Bδ, B′α, B′β, B′γ, B′δ, B′ε, B′′β, Strn and Strn3. PP2A core enzyme composed of Aα and Cα, and PP2A holoenzyme composed of Aα, Cα and various B subunits were purified from cell lysates *via* anti‐Flag (M2) agarose beads with ultra‐free Centrifugal Filter Units in the previously described buffer. Phosphorylated IKKβ proteins (0.001 μg) or protein complexes (0.001 μg) of phosphorylated IκBαRR and RelA were incubated with purified PP2A (0.001 μg) for 30 min at 37 °C, and then reaction mixtures were subjected to SDS/PAGE. Gel‐separated proteins were transferred to PVDF membranes and subjected to immunoblotting using anti‐phospho‐IKKα/β, anti‐phospho‐IκBα and anti‐phospho‐RelA antibodies, respectively.

## Results

### The PP2A core enzyme downregulates NF‐κB signalling

**Figure 1:**
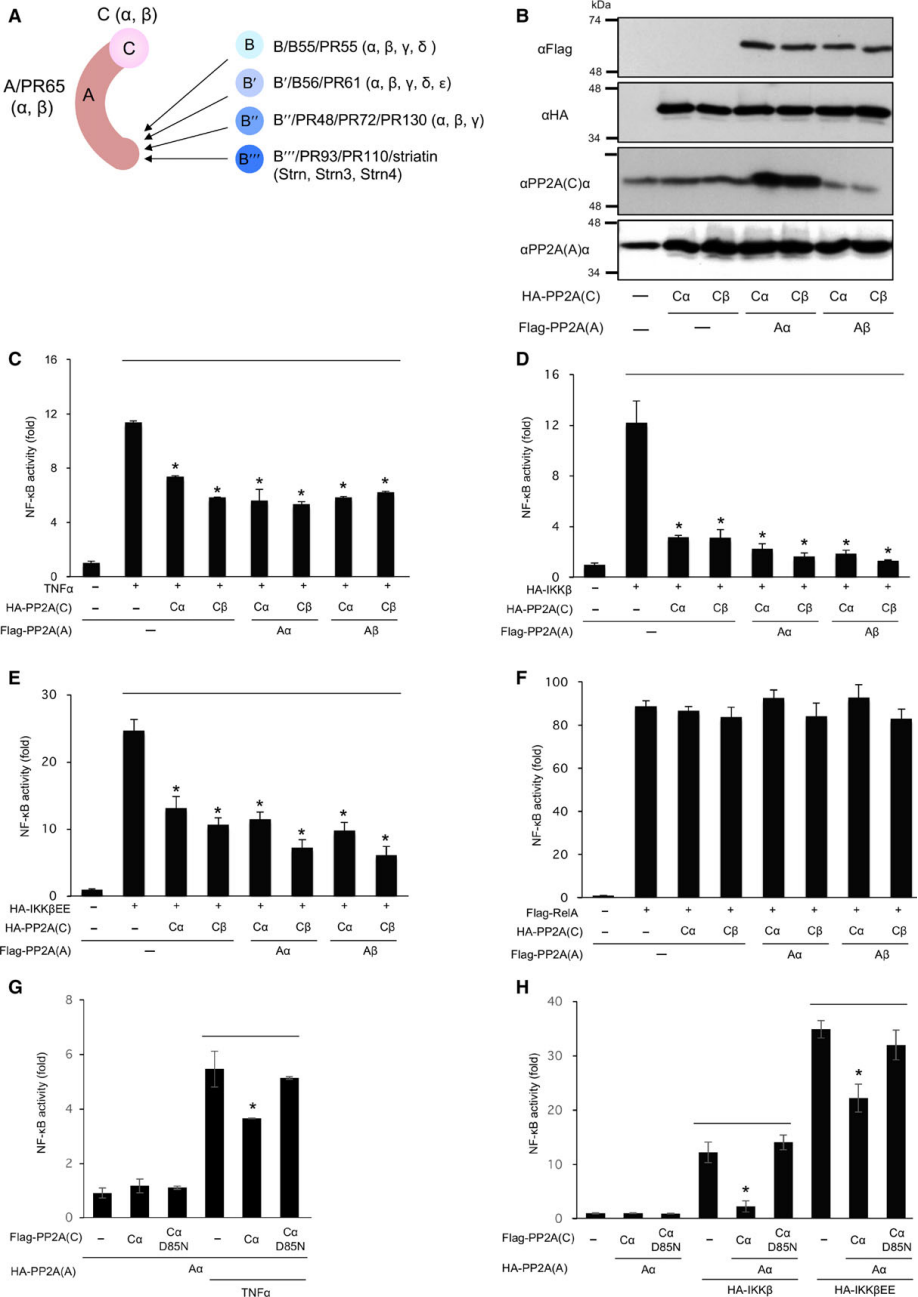
PP2A core enzymes suppress NF‐κB activity. (A) Schematic model of PP2A core enzyme and holoenzyme. (B) Expression of PP2A subunits in GP2‐293 cells. Cells were transfected with plasmids encoding HA‐PP2A Cα and Cβ, together with Flag‐PP2A Aα and Aβ, and expression of these subunits was analysed by immunoblotting. (C) Cells were transfected with NF‐κB reporter plasmids together with plasmids encoding PP2A core enzyme subunits, then stimulated with TNF‐α for 18 h. (D‐F) Cells were transfected with NF‐κB reporter plasmids and plasmids encoding PP2A core enzyme subunits together with plasmids encoding HA‐IKKβ (D), HA‐IKKβEE (E) or Flag‐RelA (F). (G) Cells were transfected with NF‐κB reporter plasmids and PP2A plasmids encoding Cα or CαD85N, and were incubated for 20 h. Then, cells were stimulated with TNF‐α for 4 h. (H) Cells were transfected with NF‐κB reporter plasmids and PP2A plasmids encoding Cα or CαD85N, together with plasmids encoding HA‐IKKβ and HA‐IKKβEE. Cells were incubated for 18 h, and NF‐κB activity was estimated by luciferase assay. *Statistically significance at *P* < 0.01.

PP2A is composed of three subunits scaffolding (A), regulatory (B) and catalytic (C) subunit (Fig. 1A). Cells were transfected with expression plasmids of α and β isoforms of the A and C subunits, and the expression of PP2A proteins were analysed by immunoblotting (Fig. 1B). Then, the effects of expression of these proteins on NF‐κB activity were analysed by luciferase assay. Expression of the Cα and Cβ subunits and of the C subunits together with Aα or Aβ subunits suppressed TNF‐α‐induced NF‐κB activation (Fig. 1C) and suppressed IKKβ and constitutive active IKKβEE‐induced NF‐κB activation (Fig. 1D,E). In contrast, expression of the PP2A did not attenuate RelA‐mediated NF‐κB activity (Fig. 1F). These results suggest that PP2A suppresses NF‐κB activity by suppressing IκBα and/or IKKβ phosphorylation. Expression of inactive PP2A core enzyme consisting of the CαD85N mutant did not suppress TNF‐α‐, IKKβ‐ and IKKβEE‐induced NF‐κB activation (Fig. 1G,H).

### Distinct PP2A holoenzymes downregulate NF‐κB signalling

**Figure 2:**
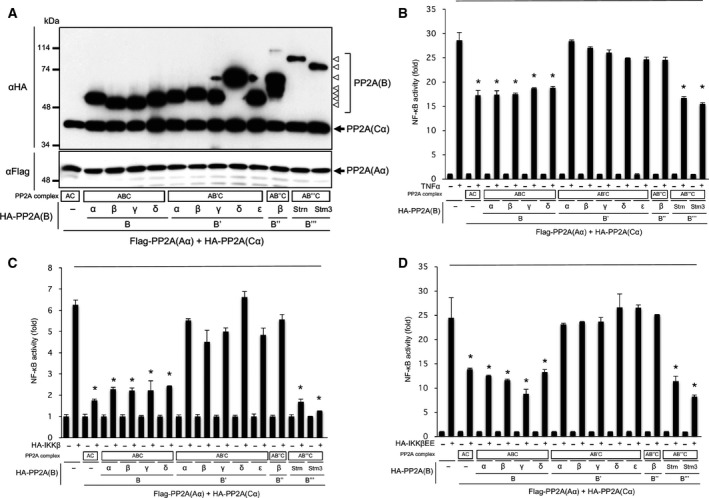
PP2A core enzyme AC and holoenzymes ABC and AB′′′C suppress TNF‐α‐ and IKKβ‐induced NF‐κB activity. (A) GP2‐293 cells were transfected with plasmids encoding PP2A core enzyme subunits Aα and Cα together with subunits of B, B′, B′′ and B′′′/Strn families. Following incubation for 24 h, cells were lysed and expression of these subunits was analysed by immunoblotting. (B) Cells were transfected with NF‐κB reporter plasmids and plasmids encoding PP2A subunits, and then stimulated with TNF‐α for 18 h. NF‐κB activity was estimated by luciferase assay. (C, D) Cells were transfected with NF‐κB reporter plasmids and plasmids encoding PP2A subunits together with plasmids encoding IKKβ (D) and IKKβEE (E). Cells were incubated for 18 h, and NF‐κB activity was estimated by luciferase assay. *Statistically significance at *P* < 0.01.

To investigate the involvement of the PP2A in regulation of NF‐κB signalling, we constructed expression plasmids encoding each subunit of the B, B′′, B′′ and B′′′/Strn family (Fig. 2A). Among isoforms of B′′ and B′′′/Strn families, we used expression plasmids encoding B′′β for the B′′ family, along with Strn and Strn3 for the B′′′/Strn family, as B′′α, B′′γ and B′′′/Strn4 hardly express in cells. Cells were transfected with NF‐κB reporter plasmids and plasmids encoding Aα, Cα and each B subunit, and NF‐κB activity was investigated following TNF‐α treatment by luciferase assay. Expression of the AC holoenzyme, the ABC holoenzyme including A(Bα)C, A(Bβ)C, A(Bγ)C and A(Bδ)C, and the AB′′′C holoenzyme including A(Strn)C and A(Strn3)C suppressed TNF‐α‐induced NF‐κB activity, whereas the AB′C holoenzyme including A(B′α)C, A(B′β)C, A(B′γ)C, A(B′δ)C and A(B′ε)C and the AB′′C holoenzyme including A(B′′β)C had little effect on TNF‐α‐induced NF‐κB activation (Fig. 2B). Then, we investigated the effects of distinct B subunits on NF‐κB signalling. NF‐κB activity was analysed in cells transfected with plasmids encoding IKKβ or IKKβEE together with Aα, Cα and each B subunit. A luciferase assay demonstrated that expression of AC, ABC and AB′′′C attenuated IKKβ‐ and IKKβEE‐induced NF‐κB activity, whereas AB′C and AB′′C had little effect on NF‐κB activation (Fig. 2C,D). These results suggest that AC, ABC and AB′′′C suppress NF‐κB activity downstream of IKKβ.

### AC and AB′′′C dephosphorylate IKKβ and AC, ABC and AB′′′C dephosphorylate IκBα in cells

**Figure 3:**
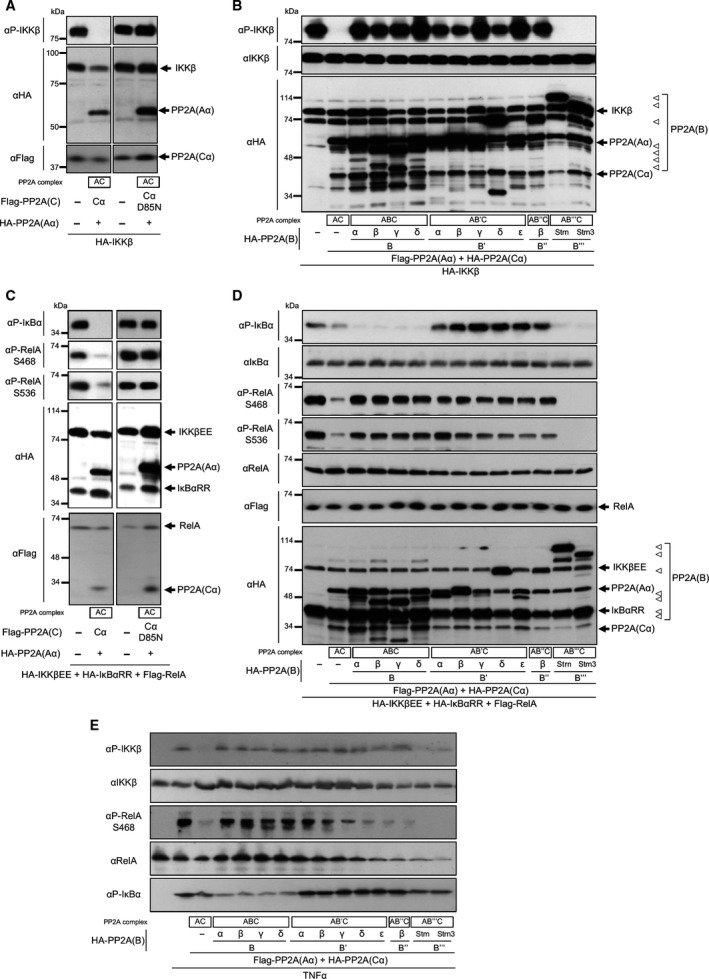
PP2A enzyme AC and AB′′′C suppress IKKβ and RelA phosphorylation, and AC, ABC and AB′′′C suppress IκBα phosphorylation *in vivo*. (A) GP2‐293 cells were transfected with plasmids encoding IKKβ and PP2A plasmids encoding Aα and Cα or phosphatase‐inactive CαD85N. (B) Cells were transfected with plasmids encoding IKKβ and PP2A plasmids encoding Aα, Cα and each subunit of B, B′, B′′ and B′′′/Strn families. (C) Cells were transfected with plasmids encoding IKKβEE, IκBαRR, RelA and PP2A plasmids encoding Aα and Cα or CαD85N. (D) Cells were transfected with plasmids encoding IKKβEE, IκBαRR, RelA and PP2A plasmids encoding Aα, Cα, and each subunit of B, B′, B′′ and B′′′/Strn families. Following incubation for 24 h, cells were lysed, and phosphorylation of proteins was estimated by immunoblotting. (E) GP2‐293 cells were transfected with expression plasmids of PP2A, and incubated for 24 h. Cells were stimulated with TNF‐α for 5 min, and phosphorylation of proteins was analysed by immunoblotting.

We next investigated the effects of distinct B subunits on phosphorylation of the IKKβ activation loop. Cells were transfected with expression plasmids encoding IKKβ and plasmids encoding Aα, Cα and each B subunit, and phosphorylation of the activation loop was investigated by immunoblotting. Expression of the AC core enzyme markedly suppressed IKKβ phosphorylation in a phosphatase activity‐dependent manner (Fig. 3A). The AC core enzyme and the AB′′′C holoenzyme markedly suppressed IKKβ phosphorylation, whereas three types of holoenzyme including ABC, AB′C and AB′′C had little effect (Fig. 3B). These lines of evidence indicate that AC and AB′′′C attenuate TNF‐α‐induced NF‐κB activity, at least in part, through dephosphorylation of IKKβ.

To reveal the effects of each B subunit on the NF‐κB/IκBα complex, we transfected cells with expression plasmids encoding IκBαRR, RelA and IKKβEE, together with plasmids encoding Aα, Cα and each B subunit. Then, the effects of PP2A on IKKβEE‐mediated phosphorylation of IκBαRR and RelA were investigated by immunoblotting. Expression of the AC core enzyme suppressed phosphorylation of IκBαRR and RelA in a phosphatase activity‐dependent manner (Fig. 3C). AC, ABC and AB′′′C reduced IκBα phosphorylation efficiently, whereas AB′C and AB′′C had little effect on dephosphorylation of IκBαRR (Fig. 3D). In turn, AC and AB′′′C dephosphorylated RelA efficiently, whereas ABC, AB′C and AB′′C had little effect on dephosphorylation of RelA. These results suggest that AC, ABC and AB′′′C attenuate NF‐κB activity, at least in part, by dephosphorylation of IκBα.

Then, we investigated the effects of distinct B subunits on phosphorylation of endogenous NF‐κB signalling proteins in TNF‐α‐stimulated cells. Cells were transfected with expression plasmids of PP2A, and then stimulated with TNF‐α. AC and AB′′′C suppressed TNF‐α‐induced phosphorylation of IKKβ and RelA, whereas ABC, AB′C and AB′′C had little effect (Fig. 3E). AC, ABC and AB′′′C reduced TNF‐α‐induced IκBα phosphorylation, whereas AB′C and AB′′C had little effect. These results suggest that functional features of B subunits have physiological significance in NF‐κB signalling.

### PP2A core enzyme and holoenzyme effectively dephosphorylate IKKβ, IκBα and RelA *in vitro*

**Figure 4:**
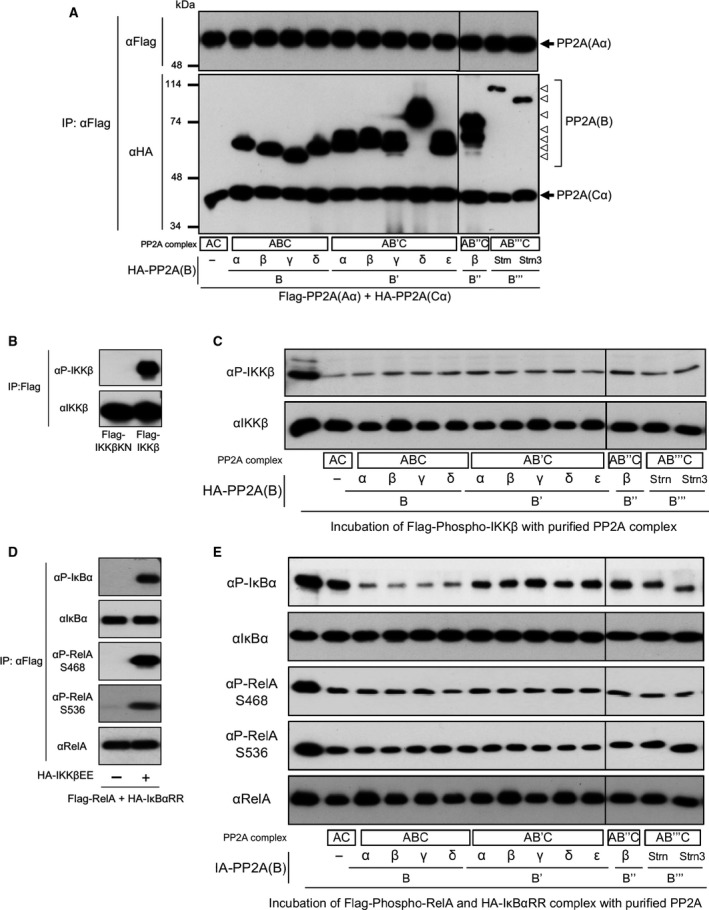
Dephosphorylation of IKKβ, IκBα and RelA by PP2A *in vitro*. (A) Cells were transfected with plasmids encoding PP2A core enzyme subunits Aα and Cα together with subunits of B, B′, B′′ and B′′′/Strn families. Following incubation for 24 h after transfection, PP2A enzymes were purified using anti‐Flag (M2) agarose beads. Purified proteins (0.01 μg) were subjected to SDS/PAGE and analysed by immunoblotting. (B) Cells were transfected with plasmids of Flag‐IKKβ and kinase‐inactive Flag‐IKKβKN. Phosphorylated IKKβ proteins were purified by using anti‐Flag (M2) agarose beads. (C) Phosphorylated IKKβ was incubated with purified PP2A, and dephosphorylation levels of IKKβ were analysed by immunoblotting. (D) Cells were transfected with plasmids encoding RelA, κBαRR and IKKβEE. Phosphorylated RelA and IκBα complexes were purified using anti‐Flag (M2) agarose beads. (E) Phosphorylated RelA and IκBα proteins were incubated with purified PP2A, and dephosphorylation levels of each protein were analysed by immunoblotting.

Dephosphorylation of IKKβ, IκBαRR and RelA was analysed by using purified PP2A enzyme *in vitro*. Expression plasmids encoding Flag‐tagged Aα, HA‐tagged Cα and HA‐tagged B subunits were transfected in cells, and PP2A complexes of various isoforms of B subunit family were purified from cells by using Flag‐agarose beads (Fig. 4A). Phosphorylated IKKβ was purified from cells transfected with Flag‐IKKβ expression plasmid (Fig. 4B). Following incubation of phosphorylated IKKβ with various purified PP2A core enzyme and holoenzyme, dephosphorylation of IKKβ was analysed by immunoblotting. This assay revealed that purified PP2A complexes of different B subunits effectively dephosphorylate IKKβ, and these complexes showed similar levels of activity (Fig. 4C). Then, we purified phosphorylated complexes of IκBαRR and RelA from cells transfected with plasmids of Flag‐RelA, HA‐IκBαRR and IKKβEE by using Flag‐agarose beads (Fig. 4D). Following incubation of phosphorylated IκBαRR and RelA protein complexes with various purified PP2A core enzyme and holoenzyme, dephosphorylation of IκBαRR and RelA was analysed by immunoblotting. Purified PP2A complexes of different B subunits effectively dephosphorylate IκBαRR and RelA (Fig. 4E). These results indicated that, although all PP2A complexes have an ability to dephosphorylate IKKβ, IκBα and RelA *in vitro*, AC and AB′′′C preferentially dephosphorylate IKKβ and RelA, and AC, ABC and AB′′′C preferentially dephosphorylate IκBα *in vivo*, suggesting that intracellular mechanisms may relate to the preference of AC, ABC and AB′′′C to dephosphorylate IKKβ, IκBα and RelA.

### Distinct B subunits regulate the binding of PP2A to the substrate proteins *in vivo*

**Figure 5:**
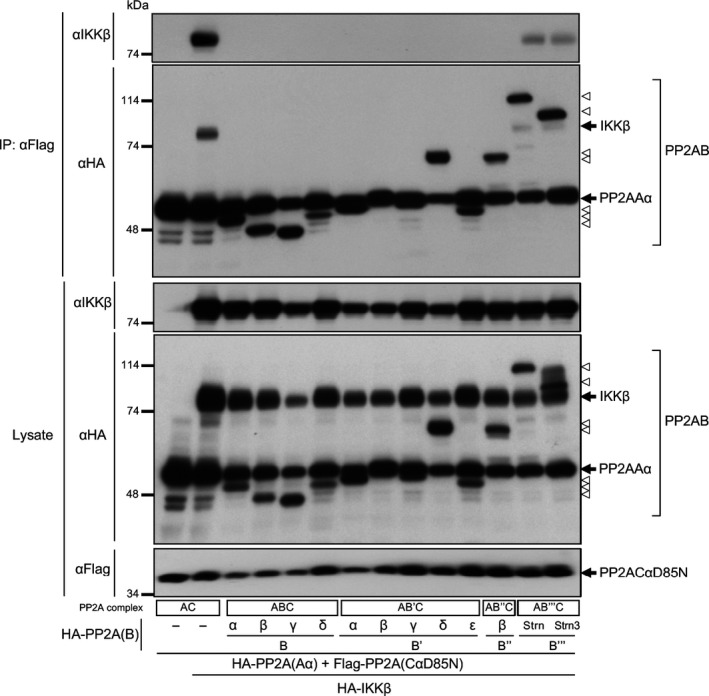
PP2A B, B′ and B′′ subunits suppress the association of PP2A AC to IKKβ *in vivo*. Cells were transfected with plasmids encoding Aα, CαD85N mutant and subunits of B families together with IKKβ plasmids. Following incubation for 24 h after transfection, PP2A complexes were immunoprecipitated and the association of IKKβ proteins to the PP2A complexes was analysed by immunoblotting.

To resolve the discrepancies in the dephosphorylation of IKKβ and RelA between *in vitro* and *in vitro,* we investigated intracellular mechanisms of the preference of AC, ABC, AB′′C and AB′′′C to dephosphorylate NF‐κB signalling proteins. The effects of B subunits on the interaction between PP2A and IKKβ were analysed by immunoprecipitation assay. Cells were transfected with plasmids encoding Flag‐tagged CαD85N, HA‐tagged Aα, HA‐tagged B subunits and HA‐tagged IKKβ. PP2A complexes were immunoprecipitated by using Flag‐agarose beads from cells, and the association of IKKβ proteins to the PP2A complexes was analysed by immunoblotting (Fig. 5). This assay revealed that IKKβ associates to PP2A, and B, B′ and B″ subunits block the association. These results suggest that the preference of AC, ABC, AB′′C and AB′′′C to dephosphorylate NF‐κB signalling proteins is regulated by the binding features of B subunits to the substrate proteins in cells.

## Discussion

Many studies have revealed important and complicated roles of PP2A in IKKβ regulation. For example, it has been reported that PP2A suppresses IKKβ activity *in vitro* and *in vivo* 7, 15, 16. However, another contradictory study shows that PP2A forms a stable complex with IKKβ and promotes its kinase activity *in vivo* 28. We found here that AC and AB′′′C dephosphorylate IKKβ in cells, whereas ABC, AB′C and AB′′C do not. These results suggest the possibility that ABC, AB′C and AB′′C complexes positively regulate IKKβ by competing against AC and AB′′′C complexes. The data in this study may help to reconcile the seemingly contradictory observations that PP2A both downregulates and promotes IKKβ activity. To confirm the specific regulation by distinct PP2A complexes, we tried to knockdown of multiple isoforms of each B family protein by RNAi. However, RNAi screen in GP2‐293 cells did not clarify distinct functions of each isoform of B family protein, because expression levels of B and B‴ family proteins are very low in GP2‐293 cells, and these cells express multiple isoforms of B′ and B″ family proteins.

NF‐κB signalling is not uniformly regulated by PP2A but rather is subjected to specific regulation by distinct PP2A complexes at multiple steps. In contrast to AC and AB′′′C, which dephosphorylate IKKβ, IκBα and RelA, ABC dephosphorylates IκBα but not IKKβ and RelA in cells. These results indicate that ABC suppresses NF‐κB activity without inhibition of IKKβ. A recent study revealed that IKKβ not only activates NF‐κB through the phosphorylation of IκBα but also regulates many cellular functions by phosphorylating various proteins in an NF‐κB‐independent manner 29. For example, IKKβ regulates autophagy 30, mRNA stability 31, apoptosis 32, angiogenesis 33 and cellular vesicle trafficking 34 by phosphorylating various proteins that are unrelated to IκBα. Thus, ABC potentially suppresses NF‐κB without inhibiting these NF‐κB‐independent functions of IKKβ, thereby ensuring that PP2A regulates NF‐κB activity and IKKβ‐mediated cellular responses independently.

Among the four classes of B subunit families, the B′′′/Strn family proteins only facilitate dephosphorylation of three proteins including IKKβ, IκBα and RelA. Members of the B′′′/Strn family are evolutionarily conserved and have critical roles in biological processes such as development and cell growth 35, 36. B′′′/Strn family proteins form a large complex termed Striatin‐interacting phosphatase and kinase (STRIPAK) along with the germinal centre kinase family and many adaptor proteins, and recruit C subunits *via* A subunits of PP2A in the complexes. STRIPAK complexes have a critical role in protein dephosphorylation and act as important regulators of multiple vital signalling pathways, including the Hippo pathway, mitogen‐activated protein kinases and cytoskeleton remodelling. Recent studies suggest that the dysregulation of STRIPAK complexes correlates with human diseases including cancer 35, 36, 37. Regulation of NF‐κB signalling pathway by AB‴C may, therefore, be involved in STRIPAK complex‐related biological processes and cancer.

PP2A is a confirmed tumour suppressor protein that is genetically altered or functionally inactivated in many cancers 22, 23, 24. Its A and C subunits are reportedly inactivated through several mechanisms including somatic mutation, phosphorylation, methylation, and/or increased expression of PP2A inhibitors, and inactivation of PP2A is linked to cancer progression. Expression of Bα, Bβ and Bγ is also decreased in many cancers owing to deletion, point mutation and DNA hypermethylation 38, 39, 40, 41, 42, 43, 44. Furthermore, it has been revealed that IKKβ and NF‐κB signalling pathways are linked to inflammation and cancer 45. Therefore, the regulation of IKKβ and NF‐κB signalling by distinct PP2A complexes may be involved in multiple human diseases including cancer, and thus might serve as specific and novel targets for disease therapy.

## Author contributions

HK and YT designed and performed the experiments, interpreted the study, and wrote the paper. KO, MK, YN, ET and TH performed the experiments.
